# Impact of adiposity on cardiac structure in adult life: the childhood determinants of adult health (CDAH) study

**DOI:** 10.1186/1471-2261-14-79

**Published:** 2014-07-01

**Authors:** Robyn J Tapp, Alison Venn, Quan L Huynh, Olli T Raitakari, Obioha C Ukoumunne, Terence Dwyer, Costan G Magnussen

**Affiliations:** 1 Melbourne School of Population and Global Health, The University of Melbourne, Parkville, Australia; 2 Department of Optometry and Vision Sciences, The University of Melbourne, 4th Floor │ Alice Hoy Building (Blg 162), Monash Road │, Melbourne, Australia; 3 Menzies Research Institute Tasmania, University of Tasmania, Hobart, Australia; 4 The Research Centre of Applied and Preventive Cardiovascular Medicine, University of Turku, and the Department of Clinical Physiology AND NUCLEAR MEDICINE, Turku University Hospital, Turku, Finland; 5 PenCLAHRC, University of Exeter Medical School, University of Exeter, Exeter, UK; 6 Murdoch Children’s Research Institute, Royal Children’s Hospital Parkville, Melbourne, Australia

## Abstract

**Background:**

We have examined the association between adiposity and cardiac structure in adulthood, using a life course approach that takes account of the contribution of adiposity in both childhood and adulthood.

**Methods:**

The Childhood Determinants of Adult Health study (CDAH) is a follow-up study of 8,498 children who participated in the 1985 Australian Schools Health and Fitness Survey (ASHFS). The CDAH follow-up study included 2,410 participants who attended a clinic examination. Of these, 181 underwent cardiac imaging and provided complete data. The measures were taken once when the children were aged 9 to 15 years, and once in adult life, aged 26 to 36 years.

**Results:**

There was a positive association between adult left ventricular mass (LVM) and childhood body mass index (BMI) in males (regression coefficient (*β*) 0.41; 95% confidence interval (CI): 0.14 to 0.67; p = 0.003), and females (*β* = 0.53; 95% CI: 0.34 to 0.72; p < 0.001), and with change in BMI from childhood to adulthood (males: *β* = 0.27; 95% CI: 0.04 to 0.51; p < 0.001, females: *β* = 0.39; 95% CI: 0.20 to 0.58; p < 0.001), after adjustment for confounding factors (age, fitness, triglyceride levels and total cholesterol in adulthood). After further adjustment for known potential mediating factors (systolic BP and fasting plasma glucose in adulthood) the relationship of LVM with childhood BMI (males: *β* = 0.45; 95% CI: 0.19 to 0.71; p = 0.001, females: *β* = 0.49; 95% CI: 0.29 to 0.68; p < 0.001) and change in BMI (males: *β* = 0.26; 95% CI: 0.04 to 0.49; p = 0.02, females: *β* = 0.40; 95% CI: 0.20 to 0.59; p < 0.001) did not change markedly.

**Conclusions:**

Adiposity and increased adiposity from childhood to adulthood appear to have a detrimental effect on cardiac structure.

## Background

Cardiac structure and functional changes are known to predict morbidity and mortality in later life and may be linked to modifiable factors in early life. Cohort studies have shown childhood risk factors influence the subsequent risk of coronary heart disease in adult life. For example, vascular endothelial dysfunction, an early stage in the development of atherosclerosis, has been demonstrated in children [1] long before any substantial risk factor burden is identifiable [2]. Cross-sectional studies of children and youth have demonstrated elevations in left ventricular mass (LVM) in association with elevated blood pressure [3], type 1 diabetes [4] and increased body mass index (BMI) [5,6]. This association is of major interest as it is well established that adults with left ventricular (LV) hypertrophy, a consequence of increased LVM, are at an increased risk of myocardial infarction, congestive heart failure and cardiovascular disease (CVD) mortality [7,8].

The few longitudinal studies to examine the association of adiposity (primarily BMI) in childhood and change in adiposity from childhood to adulthood with cardiac structure have largely allowed for the confounding effect of lipids and sex, and the mediating effect of systolic BP [9-11], these studies have not considered physical fitness or glucose metabolism as a potential confounder of the association in the primary analyses [11]. Given that diabetes exerts adverse effects on systolic and diastolic LV function independent of hypertension and coronary artery disease [12-14]; that this association is evident well below the threshold for diabetes [15]; and that fitness impacts on cardiac structure [16], assessment of these factors (both confounding and mediating) in early to mid-adulthood is warranted. Furthermore, previous longitudinal studies have used body mass index (BMI) in childhood as the measure of body size or obesity, but have not differentiated between fat and lean mass, which may have different and perhaps opposing effects on cardiac structure.

The Childhood Determinants of Adult Health study (CDAH) is a longitudinal, population-based cohort of young adults followed since childhood in Australia. This provides an important opportunity to examine the association between adiposity (potential predictor) and cardiac structure in adulthood (outcome), using a life course approach that takes account of the contribution of adiposity in both childhood and adulthood.

## Methods

### Study population

The CDAH study is a population-based, prospective cohort study established to examine childhood predictors of adult CVD and diabetes. The CDAH study design and procedures have been described in detail elsewhere but will be summarized here [17,18]. Extensive lifestyle and biological data were collected in 1985 (baseline) on a representative sample of 8498 school children aged 7 to 15 years as part of the Australian Schools Health and Fitness Survey [19]. A sub-sample of 2809 children (9, 12 and 15 year-olds) underwent additional measurements, including blood pressure, blood lipids, and further fitness tests. These additional measurements were restricted to a sub-sample owing to economic and time constraints. The follow-up study, CDAH, was performed from May 2004 to May 2006. A total of 2410 were re-measured at one of 34 field-work clinics across Australia. The population was then aged 26–36 years. During the follow-up survey, a random sample of 204 participants (approximately 1 in 3 participants with relevant childhood measures as 9, 12 and 15 year-olds) had M-mode echocardiography performed. Complete data for these analyses were available on 181 participants. Echocardiography was restricted to a sub-sample owing to time constraints of field-clinics and the need to reduce respondent burden. Participants who received echocardiography were similar to those clinic attendees who did not have the examination with respect to BMI, total cholesterol, low-density lipoprotein cholesterol (LDL-C), high-density lipoprotein cholesterol (HDL-C), triglycerides, insulin, and glucose, but had higher mean (SD) systolic (121 (13) vs 117 (13)) and diastolic (75 (9) vs 72 (9)) blood pressure, and waist circumference (86.0 (12.5) vs 83.6 (12.3)). The childhood characteristics of those with cardiac measures were also very similar to those who did not attend the follow-up study as adults on the following measures: BMI, waist circumference, total cholesterol, LDL-C, triglycerides, systolic and diastolic BP. Those with cardiac measures, however, had higher mean (SD) HDL-C (1.52 (0.31) vs 1.45 (0.28)).

At baseline, consent from both parent and child was required for inclusion in the study; at follow-up, all participants gave written informed consent. The baseline study was approved by the State Directors General of Education and the follow-up survey was approved by the Southern Tasmania Health and Medical Human Research Ethics Committee.

Weight was measured with participants in light clothes without shoes using regularly calibrated bathroom scales that recorded to the nearest 0.5 kg in 1985, and with a digital Heine portable scale that recorded to the nearest 0.1 kg at follow-up. Standing height was measured to the nearest 0.1 cm using a portable stadiometer, with the participant in bare feet. BMI was calculated using the formula: BMI = weight (kg)/height (m) [2]. Childhood overweight and obesity for BMI were defined using age and sex specific cut-points [20]. Waist circumference was measured to the nearest 0.1 cm on the skin or over light clothing. At baseline, the measurement was taken at the level of the umbilicus with a constant tension tape. At follow-up, a non-stretch tape was used to obtain the measurement at the narrowest point between the lower costal border and iliac crest.

Fat mass and percent body fat at baseline and follow-up was estimated from established regression equations that incorporate four measures of skin fold thickness. In 1985, tricep, bicep, subscapular, and suprailiac skin folds were measured at locations determined by reference to anatomical landmarks [21] using Holtain Calipers to the nearest 0.1 mm. Three readings were taken from each site, and the readings were then averaged. At follow-up, tricep, bicep, subscapular, and iliac crest skin folds were measured using Slim Guide calipers to the nearest 0.5 mm. Measurements were repeated a maximum of three times, or discontinued if the first two readings were unchanged. The average of the two closest readings was used as the location specific score. As we have previously detailed [22], skin fold values exceeding 40 mm at follow-up were imputed from BMI and waist circumference. Body density was estimated using regression equations for 9 [23], 12 and 15 year old children [24] at baseline, and for adults at follow-up [21]. Body density was then used for the calculations of fat mass and percent body fat [25].

Cardiorespiratory fitness was estimated sub-maximally at baseline and follow-up as physical working capacity at a heart rate of 170 beats per minute (PWC170) on a friction-braked bicycle ergometer (Monark Exercise AB, Sweden). The test protocol comprised three successive workloads of three minutes duration at baseline, or four minutes duration at follow-up. The workloads were selected on an individual basis to induce steady-state heart rate responses from the participant at the end of each workload. Heart rate was recorded during the final 20 seconds of each workload. Physical work capacity at 170 beats per minute was estimated by extrapolating the line of best fit from the heart rates recorded at each sub-maximal workload [26,27]. Cardiorespiratory fitness is expressed in relative terms as watts per kg (W/kg) of lean body mass.

Blood pressure measurements were obtained from the left brachial artery using a standard mercury sphygmomanometer at baseline, and from the right brachial artery using a digital automatic monitor (Omron HEM907, Omron Healthcare Inc, Kyoto, Japan) at follow-up. Blood pressure levels are reported as the mean of two measurements at baseline and the mean of three measurements at follow-up.

Blood samples were collected at baseline and follow-up from the antecubital vein after an overnight fast. In 1985, serum total cholesterol and triglycerides were determined according to the Lipids Research Clinic Program [28], and HDL-C analyzed following precipitation of apolipoprotein-B containing lipoproteins with heparin-manganese [19]. In 2004–2006, serum total cholesterol, triglyceride, and HDL-C concentrations were determined enzymatically [18]. LDL-C concentration was calculated using the Friedewald formula [29]. At follow-up only, plasma glucose levels were measured enzymatically using the Olympus AU5400 automated analyser. Two methods of insulin determination were used during the follow-up study. Plasma insulin was measured by a microparticle enzyme immunoassay kit (AxSYM, Abbot Laboratories, Abbot Park, Illinois, USA) initially, before a change in kit by the laboratory to measure serum insulin determined by electrochemiluminescence immunoassay (Elecsys Modular Analytics E170; Roche Diagnostics, Mannheim, Switzerland). Insulin levels assayed using the first methodology were corrected to levels in participants assayed using the second methodology (as per correction factor equation of the laboratory).

### Echocardiography

Two-dimensional M-mode echocardiography was performed using a portable Acuson Cypress (Siemens Medical Solutions USA Inc., Mountainview, CA) ultrasound system with a 1.8-3.6 MHz cardiac transducer by a single technician who traveled to field clinics. The left ventricle was first visualised from B-mode images obtained from the standard parasternal long-axis view. Once a clear image was obtained, the M-mode line of sight was angled perpendicular to the left ventricle between the tip of the mitral valve and papillary muscle. All images were stored on the system’s hard-drive for off-line measurement by a single technician, blinded to the participants status (obese versus non-obese). Measurements were obtained using the machine’s internal programmed calipers. End-diastolic and end-systolic measurements were made from a single cardiac cycle and then repeated on a second cardiac cycle with the measurements averaged. Cardiac measures included: inter-ventricular septum thickness (IVST), left ventricular internal diameter (LVID), left ventricular posterior wall thickness (PWT), ejection fraction, stroke volume, cardiac output and cardiac index. Images used for measurement were given a subjective rating of image quality by the technician with 1 = excellent, 2 = average, and 3 = unacceptable. Six participants had images that were graded as unacceptable and were not used for the analyses. Left ventricular mass (LVM) was calculated by the necropsy-validated formula described by Devereux: [30] LVM = {0.8 [1.04 ((IVSTd + LVIDd + PWTd)^3^ - LVIDd^3^)] + 0.6}. Left ventricular mass index (LVMI) was calculated by dividing LVM by height(m)^2.7^. Relative wall thickness (RWT) was calculated by dividing the sum of left ventricular PWT and IVST by LVID. Septal to PWT ratio was calculated by dividing IVST by PWT.

The authors of this manuscript have certified that they comply with the Principles of Ethical Publishing in the International Journal of Cardiology [31].

### Statistical methods

The data analysis was performed with Stata software (Stata Inc., 2009, Texas, USA). Means and standard deviations (or medians and interquartile ranges) were used to summarize quantitative variables and percentages were used to summarize categorical variables.

In order to provide a graphical illustration of the associations between adiposity measures and LVMI, separate categorical variables describing pattern of change between childhood and adulthood in adiposity status were created. For each adiposity measure (BMI, waist circumference, fatmass and skinfold thickness) those in the highest quartile (age and sex specific) were defined as overweight/obese. For this analysis the top quartile of each adiposity measure was used to allow direct comparison across the adiposity measures. The resulting categories of change were: (1) normal weight as child and normal weight as adult; (2) overweight/obese as child and normal weight as adult; (3) normal weight as child and overweight/obese as adult; (4) overweight/obese as child and overweight/obese as adult. We present means on the LVMI outcome for each adiposity change category.

The relationship between quantitative measures of adiposity and LVM/LVMI was estimated separately for males and females using linear regression. LVM was used to assess BMI as LVMI is indexed to height. This was undertaken as the association between adiposity and CVD risk is linear. Relationships were estimated separately for the four indicators of adiposity: BMI, waist circumference, fat mass and skinfold thickness. A life course epidemiologic approach was used that simultaneously takes account of the contribution of adiposity in both childhood and adulthood [32]. Adiposity and change in adiposity were modeled as continuous predictors in the regression models. Change is based on taking the difference of continuous scores.

Models were fitted in which childhood adiposity and change in adiposity between childhood and adulthood were used as predictors (Model 1); the first model was extended to adjust for potential confounding factors in adulthood: fitness, age, triglyceride levels and total cholesterol (Model 2); finally a third model also included potential mediating factors in adulthood: systolic BP and FPG (Model 3). In these models the regression coefficient for the child adiposity variable is the difference in the mean cardiac outcome between subjects who are one unit apart in their adiposity level at *both* childhood *and* adulthood (e.g. subject A is one unit higher in BMI than subject B at both time points). If the association were causal it would represent the effect of an increase of 1 unit in adiposity at *both* childhood *and* adulthood. The coefficient for the change in adiposity variable quantifies the mean increase in the cardiac outcome associated with a one unit difference in change (e.g. comparing cardiac outcome between two children whose *changes* in adiposity are 1 unit apart, say an increase of 2 units versus an increase of 1 unit).

## Results

Two hundred and four (204) participants underwent cardiac imaging and of these 181 had complete data available and acceptable echocardiography images. The characteristics of the participants are shown in Table 1. The mean age of participants in childhood was 12 years (range 7 to 15 years) and in adulthood 31 years (range 27 to 37 years). 54% of the sample was male. Change in BMI between participants in the sub-sample and data collected on a further 4500 participants at the time of enrollment in the follow-up study showed that the cardiac sub-sample underwent a similar mean change in BMI from childhood to adulthood to the rest of the cohort (6.8 kg/m^2^ vs. 7.1 kg/m^2^).

**Table 1:** Characteristics of the participants, at baseline in 1985 (childhood) and follow-up in 2004–6 (N = 181)

	**Males (N = 97)**		**Females (N = 84)**	
	**Childhood**	**Adulthood**	**Childhood**	**Adulthood**
	***Mean (SD)***	***Mean (SD)***	***Mean (SD)***	***Mean (SD)***
Age (years)	12 (2)	31 (3)	11 (3)	31 (3)
Height (cm)	150.6 (15.0)	179.7 (6.1)	148.5 (14.1)	167.7 (6.1)
Weight (kg)	42.7 (13.2)	86.1 (13.9)	41.0 (11.7)	72.8 (18.0)
BMI (kg/m^2^)	18.3 (2.4)	26.6 (3.5)	18.2 (2.6)	25.9 (6.1)
Waist circumference (cm)	65.4 (7.7)	90.3 (9.2)	62.2 (7.1)	80.5 (13.6)
Skin fold thickness (mm)*	40.2 (19.8)	67.1 (25.5)	54.2 (21.9)	88.4 (34.6)
Fitness (W/kg)	3.1 (0.6)	3.0 (0.6)	2.7 (0.7)	3.1 (0.7)
Systolic BP (mmHg)^†^	108.5 (14.7)	127.9 (10.3)	108.7 (14.1)	113.1 (10.1)
Diastolic BP (mmHg)^†^	64.6 (12.0)	77.8 (8.3)	67.1 (10.8)	72.0 (9.0)
Total cholesterol (mmol/l)^‡^	4.4 (0.8)	5.0 (0.9)	4.6 (1.2)	4.8 (1.2)
HDL cholesterol (mmol/l)^‡^	1.4 (0.4)	1.3 (0.3)	1.5 (0.5)	1.5 (0.3)
LDL cholesterol (mmol/l)^‡^	2.6 (0.6)	3.1 (0.7)	2.8 (0.6)	2.9 (1.1)
Triglycerides (mmol/l)^‡^	0.7 (0.49 - 0.8)	1.1 (0.7 - 1.6)	0.7 (0.6 - 0.8)	0.8 (0.6 - 1.2)
Fasting plasma glucose (mmol/l)	¥	5.2 (0.4)	¥	4.9 (0.4)
Fasting Insulin	¥	7.4 (4.5)	¥	7.3 (4.5)

Data given as mean (S.D.) or median and (inter-quartile range) *Number (N) of boys = 80 and girls = 72 in childhood. ^†^N = 80 for boys and 68 for girls systolic and 67 for girls diastolic in childhood, ^‡^HDL cholesterol and LDL cholesterol: 66 boys and 51 girls in childhood, total cholesterol and triglycerides: 67 boys and 52 girls in childhood, ¥not measured in childhood. *Categories of overweight and obese were calculated using age and sex specific cut-points in childhood as is standard practice and according to the international standard definition in adulthood (overweight BMI ≥25 and obese ≥30).*

**Figure 1:**
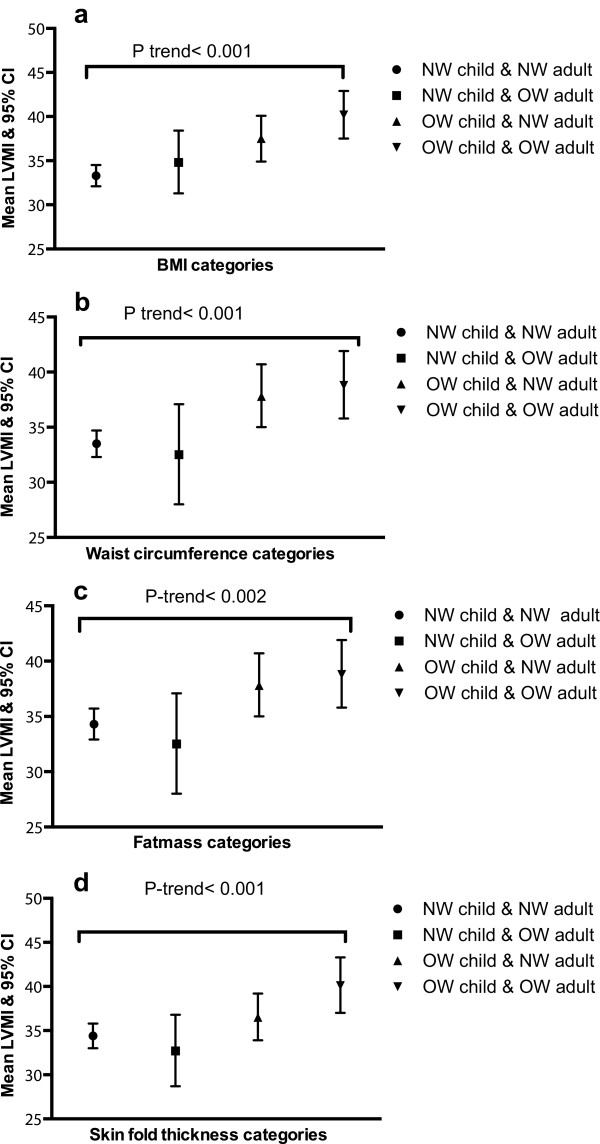
**Mean LVMI and 95% CI by categories of adiposity measures. (a)** BMI categories **(b)** Waist circumference categories **(c)** Fatmass categories **(d)** Skin fold thickness categories.

Table 2 shows the echocardiographic measures by childhood and adulthood BMI category. The table is presented combined for males and females, due to the small number of overweight and obese children (n = 16). In childhood, a pattern of higher values was observed among those in the overweight or obese category for LVM, LVMI, LV diameter in diastole and systole and PWT in systole and diastole compared with those with a BMI within the normal range. In adulthood those in the overweight and obese category had a higher LVM, LVMI, LV diameter in diastole and systole, PWT and IVST. Figures 1a to d show the mean (age and sex specific) LVMI and 95% CI by categories of change in BMI, waist circumference, fat mass and skinfold thickness, respectively. The overall p-value for each adiposity measure by LVMI was significant at the 5% level. For each adiposity measure, (BMI, waist circumference, fat mass and skin fold thickness) there was an indication of higher LVMI in those who were overweight in both childhood and adulthood or only overweight in childhood.

**Table 2:** Echocardiographic measures of the participants by BMI category in each of childhood and adulthood

	**Childhood BMI**		**Adult BMI**	
	**Normal weight**	**Overweight/Obese**	**Normal weight**	**Overweight/Obese**
N	165	16	81	100
Left ventricular mass (g)	155.9 (39.9)	182.5 (40.4)	139.3 (34.1)	173.5 (38.9)
Left ventricular mass index (g/m^2.7^)	34.6 (7.0)	39.9 (7.9)	31.8 (6.1)	37.8 (6.9)
LV diameter in diastole (cm)	4.86 (0.44)	5.18 (0.37)	4.73 (0.43)	5.02 (0.42)
LV diameter in systole (cm)	3.24 (0.42)	3.43 (0.36)	3.18 (0.43)	3.34 (0.37)
Posterior wall thickness, diastole (cm)	0.90 (0.11)	0.95 (0.09)	0.86 (0.11)	0.94 (0.10)
Posterior wall thickness, systole (cm)	1.54 (0.23)	1.63 (0.25)	1.46 (0.23)	1.60 (0.21)
Intra-ventricular septum, diastole (cm)	0.92 (0.13)	0.95 (0.11)	0.87 (0.11)	0.97 (0.13)
Intra-ventricular septum, systole (cm)	1.34 (0.26)	1.38 (0.31)	1.26 (0.25)	1.41 (0.24)
Relative wall thickness	0.38 (0.05)	0.37 (0.04)	0.37 (0.05)	0.38 (0.05)
IVSD / PWT ratio	1.02 (0.09)	1.00 (0.07)	1.01 (0.10)	1.03 (0.09)

Overweight and obesity in childhood defined by Cole et al. [33]. Data are mean and SD.

The results from the linear regressions of adult echocardiographic measures (LVM and LVMI on adiposity are shown in Table 3. LVM was used in these models when assessing BMI. In both males and females, there was a positive association between childhood BMI and LVM and a positive association between change in BMI and LVM (model 1).

**Table 3:** Multiple regression of echocardiographic measures in adulthood on measures of adiposity in childhood and change in adiposity from childhood to adulthood

		**Males**						**Females**					
		**Model 1**			**Model 2**			**Model 1**			**Model 2**		
***Body mass index (BMI)***		**Regression coefficient**	***95% CI***	***P***	**Regression coefficient**	***95% CI***	***P***	***Regression coefficient***	***95% CI***	***P***	**Regression coefficient**	***95% CI***	***P***
LVM (g)	Childhood BMI	0.46	0.23 to 0.70	<0.001	0.41	0.14 to 0.67	0.003	0.44	0.30 to 0.58	<0.001	0.53	0.34 to 0.72	<0.001
	Change in BMI	0.30	0.08 to 0.52	0.009	0.27	0.04 to 0.51	0.023	0.29	0.14 to 0.44	<0.001	0.39	0.20 to 0.58	<0.001
***Waist circumference (WC)***													
LVMI (g/m^2.7^)	Childhood WC	0.04	−0.01 to 0.09	0.10	0.04	−0.02 to 0.09	0.213	0.10	0.06 to 0.14	<0.001	0.13	0.08 to 0.18	<0.001
	Change in WC	0.04	−0.00 to 0.08	0.072	0.04	−0.01 to 0.08	0.088	0.08	0.04 to 0.13	<0.001	0.13	0.07 to 0.18	<0.001
***Fat mass (FM)***													
LVMI (g/m^2.7^)	Childhood FM	0.04	−0.01 to 0.10	0.109	0.04	−0.02 to 0.10	0.223	0.11	0.06 to 0.16	<0.001	0.15	0.09 to 0.22	<0.001
	Change in FM	0.03	−0.02 to 0.07	0.287	0.03	−0.02 to 0.08	0.237	0.07	0.03 to 0.12	0.002	0.11	0.05 to 0.16	<0.001
***Skin fold thickness (SFT)***													
LVMI (g/m^2.7^)	Childhood SFT	0.02	−0.03 to 0.07	0.462	0.01	−0.06 to 0.07	0.827	0.09	0.04 to 0.15	<0.001	0.13	0.06 to 0.20	<0.001
	Change in SFT	0.00	−0.04 to 0.05	0.901	0.01	−0.05 to 0.06	0.844	0.06	0.02 to 0.11	0.009	0.10	0.04 to 0.16	0.002

Model 1 - contains childhood adiposity and change in adiposity as predictors. Model 2 - as Model 1 but adjusted for confounding factors in adulthood: age, fitness, triglycerides and total cholesterol). LVM - Left ventricular mass. BMI, FM and LM data are ranked by age (separately in males and females) to account for the impact of age on growth among children.

Categories of overweight and obese for BMI were calculated using age and sex specific cut-points in childhood and according to the international standard definition in adulthood (overweight BMI ≥25 and obese ≥30). The highest quartile of each childhood and adult measure of adiposity (waist circumference, skin fold thickness and fatmass) measured separately in males and females) were used to define overweight and obese (age and sex specific).

Adjustment for confounding factors (in adulthood: age, fitness, triglycerides and total cholesterol), had little impact on the estimated regression coefficients (Model 2). This association did not change with adjustment for both age in childhood and adulthood or stratification by age group, with the exception of change in skinfold thickness and fat mass in females aged 11 to 15 years and BMI change in males aged 7–10 years, which were no longer significant.’ These relationships also changed little after further adjustment for mediating factors from adulthood (systolic BP and FPG, Model 3). In Model 3 the coefficient for BMI in childhood for males was 0.45 (95% CI: 0.19 to 0.71; p = 0.001) and for females was 0.49 (95% CI: 0.29 to 0.68; p < 0.001). The coefficient for change in BMI for males was 0.26 (95% CI: 0.04 to 0.49; p = 0.02) and for females was 0.40 (95% CI: 0.20 to 0.59; p < 0.001). Analyses that replaced BMI with waist circumference, fat mass or skinfold thickness in separate analyses also showed evidence of associations with LVMI in females (Table 3). When direct measures of fat (skinfold thickness and fatmass) were used the associations were much weaker than for BMI and only significant in females.

The models were additionally re-run replacing FPG with fasting insulin to assess the potential mediating impact of insulin on LVM. There was little change in the association of BMI with LVM after adjustment for the mediating impact of insulin: the regression coefficient for BMI in childhood for males was 0.47 (95% CI: 0.20 to 0.73; p = 0.001) and for females was 0.49 (95% CI: 0.29 to 0.69; p < 0.001). The coefficients for change in BMI for males was 0.28 (95% CI: 0.02 to 0.55; p = 0.04) and for females was 0.40 (95% CI: 0.20 to 0.59; p < 0.001).

## Discussion

The present study determined that childhood adiposity and change in adiposity (using several different measures of adiposity) from childhood to adulthood is associated with cardiac structure. The association with change in adiposity was independent of childhood adiposity. While previous research has suggested an association between adiposity and LVM in adolescence [34-36] and in adulthood [9-11], only one previous study has had more than 14 years of follow-up allowing assessment of the association into the third decade of life [11].

This study extends the work of the few longitudinal studies undertaken by assessing four measures of adiposity (BMI, waist circumference, fat mass and skin fold thickness, in separate models), taking a life course approach and allowing for key potential factors that might introduce confounding on these associations, separately in males and females. Overall, our results suggest that adiposity in childhood and adulthood and change in adiposity between childhood and adulthood, are associated with LVMI in adulthood. In particular, when direct measures of fat (skinfold thickness and fatmass) are used the associations were much weaker than for BMI and only significant in females, which may be related to sex hormones [37]. The risk of increased LVM/LVMI appears to be greatest among those who are overweight in childhood and adulthood, supporting the results of earlier studies.

The few longitudinal studies to examine the association of adiposity in childhood and change in adiposity from childhood to adulthood with cardiac structure have largely allowed for the confounding effect of lipids and sex, and the mediating effect of systolic BP. These studies have not considered physical fitness as a potential confounder of the association in the primary analyses [11]. The Bogalusa Heart study with 21 years of follow-up assessed cardiac structure in a subgroup of 467 young adults aged 20–38 years (mean age 32.6 years) [11]. The study showed that those with a higher BMI in childhood had larger LVM 21 years later, and those with a higher BMI in both childhood and adulthood had the largest cardiac size, with adjustment for lipids (HDL and LDL cholesterol and triglycerides) and blood pressure. The Bogalusa Heart study similarly found no mediating role for fasting glucose when assessed at a mean age of 19 years [38], with an association between diabetes and left ventricular hypertrophy evident at the 24 year follow-up [39].

In a 10 year follow-up study of African American and European American youth (mean age at baseline 14 years) with a positive family history of CVD, Dekkers et al. [9] found an increased BMI was a strong predictor of LVM. The study assessed a number of important risk factors (including SBP, pulse pressure, heart rate, ethnicity and father’s education). A further study undertaken by Sivanandam et al. [10] of 132 healthy children, at a mean age of 13 years and re-evaluated 14 years later showed that adiposity and LVM were related in childhood and that the greater the increase in BMI from childhood through to adult life the greater the increase in LVM. This study investigated the association of LVM with glucose and fasting insulin after adjustment for BMI and showed no association. Studies in adolescent and adult populations have shown mixed results for glycaemia, largely as a consequence of assessing small samples with limited power to detect associations. In the present study there was little evidence of fasting plasma glucose being associated with increased LVM or other measures of cardiac structure. Fitness is known to impact on cardiac structure [16]. In this sample adjusting for fitness did not reduce the association of adiposity with cardiac measures.

Increased adiposity is directly linked to other CVD risk factors. It is well-established that as adiposity increases so too do other CVD risk factors, i.e. blood pressure and total cholesterol. Elevated blood pressure related to obesity increases the heart’s workload and leads to cardiac growth. In the present population, blood pressure did not mediate the association of adiposity (for any of the adiposity measures) with cardiac structure. There are several mechanisms linking adiposity with adverse changes in cardiac structure and function. Of particular interest is research providing a direct link between increased adiposity and cardiac growth. It is well acknowledged that central fat is metabolically active and leads to the activation of a series of pathophysiologic processes including activation of the renin-angiotensin system and development of insulin resistance [40]. Several studies have demonstrated an association between adiposity and cardiac structure among those who are obese but not hypertensive and have shown that chronic volume overload (increased preload) and the related increased cardiac output may stimulate cardiac growth [41]. This interpretation has been supported by a number of studies and appears to be supported by the present study, as the impact of adiposity on cardiac measures was not explained by BP.

The present study has several limitations including its small sample size. Further work should be undertaken on a large population-based cohort with long follow-up, as understanding the cardiac impacts will provide insights into the likely trajectory of development of cardiac disease in individuals who are overweight or obese. It may also provide valuable information on the differences in the rate of progress of components of the pathogenic process when obesity and insulin resistance occur at an early age. Bias due to subject selection is unlikely to explain the current study results. For example, the baseline population characteristics of those with cardiac data at follow-up were similar to those who did not attend follow-up, with the exception of HDL-C. HDL-C is not an established risk factor for change in cardiac structure or function, so we do not expect this difference to have impacted on our findings. Moreover, because echocardiographs were not collected in childhood, we were unable to discount that children with increased adiposity had larger hearts already in childhood. Finally, any sex differences need to be further confirmed in studies with larger sample sizes.

## Conclusions

The impact of childhood risk factors on cardiac structure is not fully understood. This study has shown that childhood and adulthood adiposity and increased adiposity from childhood to adult life are associated with poorer cardiac structure in adulthood independent of other known factors that impact on cardiac structure. This suggests that changes in cardiac structure begin to develop in youth as a consequence of obesity.

## Competing interests

The authors declare that they have no competing interests.

## Authors’ contributions

RT undertook the data analyses, interpretation of the data, drafting of the manuscript and giving final approval of the version to be published. AV participated in the study design, interpretation of the analyses, revising the manuscript and giving final approval of the version to be published. LH assisted with the data analyses, revising the manuscript and giving final approval of the version to be published. OR assisted with interpretation of the data, drafting of the manuscript and giving final approval of the version to be published. OU assisted with interpretation of the data, drafting of the manuscript and giving final approval of the version to be published. TD participated in the study design, interpretation of the analyses, revising the manuscript and giving final approval of the version to be published. CM assisted with the interpretation of the data, drafting of the manuscript and giving final approval of the version to be published. All authors read and approved the final manuscript.

## Pre-publication history

The pre-publication history for this paper can be accessed here:

http://www.biomedcentral.com/1471-2261/14/79/prepub
